# The roles of lipid and glucose metabolism in modulation of β-amyloid, tau, and neurodegeneration in the pathogenesis of Alzheimer disease

**DOI:** 10.3389/fnagi.2015.00199

**Published:** 2015-10-23

**Authors:** Naoyuki Sato, Ryuichi Morishita

**Affiliations:** ^1^Department of Clinical Gene Therapy, Graduate School of Medicine, Osaka UniversitySuita, Japan; ^2^Department of Geriatric Medicine, Graduate School of Medicine, Osaka UniversitySuita, Japan

**Keywords:** cholesterol, diabetes, Aβ, tau, neurodegeneration

## Abstract

Diabetes is a risk factor for Alzheimer disease (AD). Apolipoprotein E (ApoE) and several genes related to AD have recently been identified by genome-wide association studies (GWAS) as being closely linked to lipid metabolism. Lipid metabolism and glucose-energy metabolism are closely related. Here, we review the emerging evidence regarding the roles of lipid and glucose metabolism in the modulation of β-amyloid, tau, and neurodegeneration during the pathogenesis of AD. Disruption of homeostasis of lipid and glucose metabolism affects production and clearance of β-amyloid and tau phosphorylation, and induces neurodegeneration. A more integrated understanding of the interactions among lipid, glucose, and protein metabolism is required to elucidate the pathogenesis of AD and to develop next-generation therapeutic options.

## Introduction

**Figure 1:**
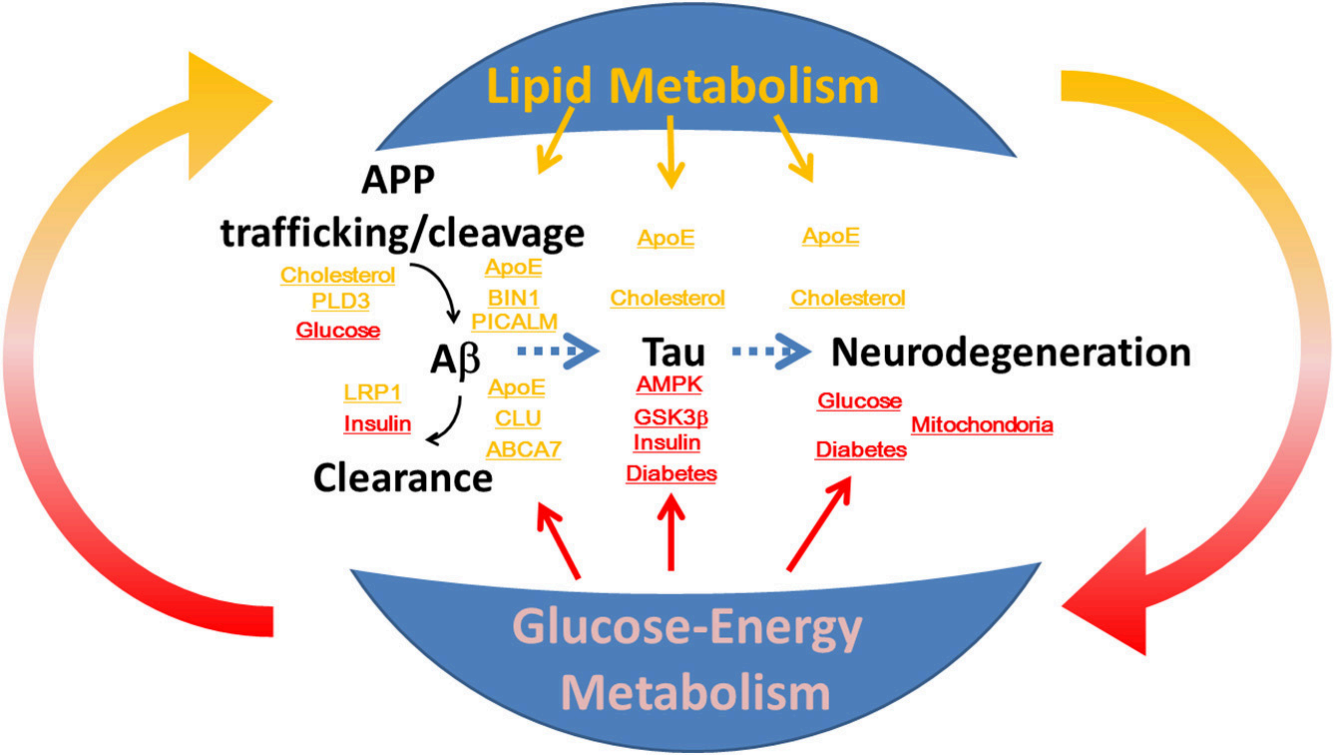
**The roles of lipid and glucose metabolism in the modulation of β-amyloid and tau during neurodegeneration and the pathogenesis of Alzheimer disease**. Disruption of homeostasis of lipid and glucose metabolism affects production and clearance of β-amyloid and tau phosphorylation, and induces neurodegeneration.

Alzheimer disease (AD) is a progressive neurodegenerative disorder that is pathologically characterized by cerebral atrophy (particularly within the hippocampus and temporal and parietal lobes), senile plaques, neurofibrillary tangles (NFT), and neuronal cell death. Familial AD is caused by mutations in the amyloid precursor protein (Goate et al., 1991) and presenilin (Sherrington et al., 1995). These mutations cause overproduction of β-amyloid (Aβ), particularly its longer form, Aβ42, which aggregates *in vitro* (Jarrett et al., 1993) and forms the initial deposits in the brain (Iwatsubo et al., 1994) to form senile plaques. Apolipoprotein E (ApoE) is an essential regulator of brain cholesterol metabolism and is the strongest genetic risk factor for sporadic AD (Ashford, 2004). In addition to the ApoE gene, recent genome-wide association studies (GWAS) have identified novel risk genes for AD (Hollingworth et al., 2011; Olgiati et al., 2011), and some of these genes are closely associated with lipid metabolism. Moreover, numerous epidemiological studies have demonstrated that patients with diabetes in which glucose-energy metabolism is affected have a significantly higher risk of developing AD (Ott et al., 1999; Kopf and Frölich, 2009; Maher and Schubert, 2009; Matsuzaki et al., 2010). However, the roles of lipid metabolism and glucose-energy metabolism in the pathogenesis of AD are not fully understood (Figure 1). Here, we review the roles of lipid and glucose metabolism in modulating Aβ, tau, and neurodegeneration during the pathogenesis of AD (Table 1) and focus on novel therapy development.

**Table 1:** **Lipid and glucose metabolism are associated with the modulation of β-amyloid and tau and neurodegeneration**.

		**Aβ**			**Tau**	**Neurodegeneration/dementia**
		**APP/Aβ trafficking/cleavage**	**Aβ clearance**			
			**Degradation /uptake**	**Phagocytic clearance**		
Lipid Metabolism	Cholesterol	+; Marquer et al., 2014; Reed et al., 2014			+; Michikawa, 2006; Ohm and Meske, 2006; Maccioni et al., 2010; Glöckner and Ohm, 2014; Shibuya et al., 2015	+; Saher and Stumpf, 2015
	APOE	+; Irizarry et al., 2004	+; Verghese et al., 2013	+; Koistinaho et al., 2004	+; Ohm et al., 1999	+; Belinson et al., 2008
	BIN1	+; Itoh and De Camilli, 2006		+; Chapuis et al., 2013	+; Chapuis et al., 2013; Tan et al., 2013; Holler et al., 2014; Zhou et al., 2014	
	CLU		+; Yu and Tan, 2012			
	ABCA7			+; Kim et al., 2013		
	PICALM	+; Kanatsu et al., 2014				
	TREM2			+; Wang et al., 2015		
	PLD3	+; Cruchaga et al., 2014				
	LRP1		+; Kanekiyo et al., 2013; Ramanathan et al., 2015			
	Statin	+; Shinohara et al., 2010	+; Shinohara et al., 2010	+; Tanaka et al., 2011a,b	+; Boimel et al., 2009; Lu et al., 2010	
GLUCOSE Metabolism	Diabetes				+; Liu et al., 2009; Sato and Morishita, 2014	+; Sato and Morishita, 2014
	Glucose	+; Macauley et al., 2015				+; Sato and Morishita, 2014; Zhu et al., 2014; Weinstein et al., 2015; Winkler et al., 2015
	Insulin		+; Vekrellis et al., 2000; Shiiki et al., 2004		+; Starks et al., 2015	
	AMPK				+; Mairet-Coello et al., 2013	+; Mairet-Coello et al., 2013
	GSK3β				+; Hanger et al., 1992; Yang et al., 1993	
	Pioglitazone		+; Mandrekar-Colucci et al., 2012	+; Mandrekar-Colucci et al., 2012		+; Heneka et al., 2015
	Metformin				+; Kickstein et al., 2010	+; Moore et al., 2013; Ng et al., 2014

## The role of lipid metabolism in β-amyloid modulation

ApoE is an essential regulator of cholesterol metabolism and is the strongest genetic risk factor for AD (Ashford, 2004). The ApoEε4 allele increases the accumulation of senile plaques in patients with AD and in cognitively normal people (Reiman et al., 2009; Morris et al., 2010). Physiologically, ApoE is important for brain function through lipid transport of high-density lipoprotein-like particles (Raber et al., 2000; Ji et al., 2003; Bu, 2009; Filippini et al., 2009; Trachtenberg et al., 2011; Verghese et al., 2011; Wisdom et al., 2011). ApoE isoforms are believed to differentially regulate Aβ aggregation and uptake, degradation, and phagocytic clearance in the brain, and each isoform has distinct functions in regulating brain lipid transport, glucose metabolism, and mitochondrial function (Koistinaho et al., 2004; Verghese et al., 2013).

Moreover, independently of ApoEε4, high levels of low-density lipoprotein cholesterol and low levels of high-density lipoprotein cholesterol are associated with higher amyloid-PET indices (Reed et al., 2014). This observation may be partially explained by an *in vitro* experiment in which transient membrane cholesterol loading increased Aβ42 secretion (Marquer et al., 2014). Interestingly, lipids also trigger the aggregation of α-synuclein, a major component of Lewy bodies, by stimulating nucleation (Galvagnion et al., 2015). This evidence increases the possibility that lipids might promote protein aggregation.

In addition to the ApoE gene, recent GWAS studies have identified novel risk genes for AD (Hollingworth et al., 2011; Olgiati et al., 2011). These genes include bridging integrator 1 (BIN1), clusterin (CLU, also called apolipoprotein J), ATP-binding cassette transporter A7 (ABCA7), triggering receptor expressed on myeloid cells 2 (TREM2), and phosphatidylinositol-binding clathrin assembly protein (PICALM). CLU is a primary brain cholesterol transport lipoprotein and may behave similarly to ApoE (Calero et al., 1999; Yu and Tan, 2012). ABCA7 is involved in lipid homeostasis (Tanaka et al., 2011b). Deletion of ABCA7 increases Aβ accumulation in amyloid precursor protein transgenic (APP Tg) mice through reduced phagocytic clearance of Aβ (Kim et al., 2013). TREM2 regulates the microglial response through lipid sensing around the senile plaque in an AD mouse model (Wang et al., 2015). BIN1 is involved in endocytosis and membrane trafficking (Itoh and De Camilli, 2006) through phosphatidylinositol binding (Lee et al., 2002; Kojima et al., 2004) and also modulates APP trafficking in neurons (Chapuis et al., 2013). BIN1 expression is also observed in microglia, suggesting a role for BIN1 in Aβ phagocytosis (Chapuis et al., 2013). PICALM also plays important roles in clathrin-mediated endocytosis (Dreyling et al., 1996) through phosphatidylinositol binding (Ford et al., 2001), suggesting involvement in APP trafficking (Xiao et al., 2012). Indeed, γ-secretase is endocytosed in a PICALM-dependent manner (Kanatsu et al., 2014). Interestingly, altering the rate of clathrin-mediated endocytosis of γ-secretase increases Aβ42 production (Kanatsu et al., 2014). ApoE also could modulate γ-secretase cleavage of APP, though all three isoforms of ε2, ε3, and ε4, have similar effects (Irizarry et al., 2004). Recent whole-exome sequencing and functional data indicate that carriers of PLD3 (phospholipase D3) coding variants have a two-fold increased risk for late-onset AD and that PLD3 influences APP processing (Cruchaga et al., 2014).

Although clinical studies have indicated that statins have no beneficial effect on cognitive function (McGuinness et al., 2014), statins administered in midlife might prevent AD in late life by modifying the genetic and non-genetic risk factors for AD (Sato et al., 2012; Shinohara et al., 2014). In experimental settings *in vivo* and *in vitro*, statins reduced the Aβ level in the brain (Fassbender et al., 2001; Burns et al., 2006; Ostrowski et al., 2007; Kurinami et al., 2008; Papadopoulos et al., 2014). Aβ reduction by a statin is associated with a reduction in the carboxyl terminal fragment of amyloid precursor protein (APP-CTF; Shinohara et al., 2010). Statins reduce the brain Aβ levels by increasing APP-CTF trafficking through isoprenylation inhibition. Moreover, a statin up-regulated Aβ clearance by up-regulating low-density lipoprotein receptor-related protein 1 (LRP1) expression in the vasculature (Shinohara et al., 2010). LRP1 play roles in the efflux of Aβ from the brain (Ramanathan et al., 2015) and neuronal Aβ uptake and degradation (Kanekiyo et al., 2013). Finally, statins enhance ABCA7-dependent phagocytosis (Tanaka et al., 2011a). Thus, lipid metabolism is involved in modulating Aβ levels.

## The role of lipid metabolism in tau modulation

Normal tau promotes the assembly and stabilization of microtubules. However, hyperphosphorylated tau sequesters normal tau and disrupts microtubules, forming NFT (Iqbal et al., 1994, 2009). In mice expressing mutant human tau, cellular cholesterol levels were higher in neurons affected by tau pathology (Glöckner and Ohm, 2014). Indeed, impaired cholesterol metabolism is involved in tau hyperphosphorylation (Michikawa, 2006; Ohm and Meske, 2006; Maccioni et al., 2010). Emerging data suggest that BIN1 modulates tau pathology in addition to Aβ (Chapuis et al., 2013; Tan et al., 2013; Holler et al., 2014; Zhou et al., 2014). BIN co-localizes and interacts with tau (Chapuis et al., 2013; Zhou et al., 2014). Therefore, BIN1 levels may correlate with NFTs in AD (Glennon et al., 2013; Holler et al., 2014). In addition to their effects on Aβ metabolism, statins suppress tau hyperphosphorylation induced by excess cholesterol in the brain (Lu et al., 2010) and also reduce NFTs in a tau pathology model (Boimel et al., 2009). Moreover, inhibition of cholesterol metabolism by blocking acyl-coenzyme A:cholesterol acyltransferase 1 activity reduces the amount of mutant human tau in neurons of triple transgenic mice (Shibuya et al., 2015). In comparison with ApoEε3, the presence of the ApoEε4 is reported to be associated with NFT formation (Ohm et al., 1999). These studies demonstrate that the regulation and dysregulation of cholesterol metabolism affect tau pathology in the brain.

## The role of lipid metabolism in neurodegeneration modulation

As the largest pool of cholesterol resides in neuronal myelin membranes, disorders that impair sterol synthesis or intracellular trafficking of lipids in neurons cause hypomyelination and neurodegeneration (Saher and Stumpf, 2015). Glial lipid droplets induced by mitochondrial defects also promote neurodegeneration (Liu et al., 2015) suggesting a role for lipid metabolism in glial cells in neurodegeneration. Moreover, the human cortex demonstrates membrane protein oxidation (Granold et al., 2015) and altered phospholipid components during aging (Norris et al., 2015). Other than AD, impaired lipid metabolism has been reported in several neurodegenerative diseases. The huntingtin gene, which is causative for Huntington disease, also seems to play a regulatory role in lipid metabolism (Leoni and Caccia, 2015). Cholesterol metabolism impairment is proportion to the CAG repeat length and to the load of mutant huntingtin leading to neurodegeneration (Leoni and Caccia, 2015). Mucopolysaccharidosis III type C, a progressive neurological pediatric disease, is caused by mutations in the heparan-α-glucosaminide N-acetyltransferase gene and leads to a deficiency in acetyl-CoA: α-glucosaminide N-acetyltransferase (Martins et al., 2015). These results further support the role of lipid metabolism in neurodegeneration. Although reversible, statins might transiently impair cognitive function, especially during the initial administration to patients older than 75 years (Orsi et al., 2001; King et al., 2003; Wagstaff et al., 2003). This effect is probably due in part to cholesterol's modulation of NMDA receptor function (Korinek et al., 2015). Finally, activation of Aβ cascade in ApoEε4 transgenic mice induces lysosomal activation and neurodegeneration resulting in marked cognitive deficits (Belinson et al., 2008). Taken together, these reports indicate that cholesterol metabolism is tightly linked to neurodegeneration.

## The role of glucose-energy metabolism in β-amyloid modulation

Diabetes in midlife is associated with mild cognitive impairment (MCI; Roberts et al., 2014), and impaired glycemia increases the disease progression to dementia in patients with MCI (Morris et al., 2014). However, the mechanisms by which diabetes modifies cognitive function remain unclear (Sato and Morishita, 2013a,b). Diabetes seems to alter brain structure and function through Aβ/tau-dependent and independent mechanisms (Sato and Morishita, 2014). Insulin resistance in midlife is associated with neurodegeneration surrounding senile plaques (Matsuzaki et al., 2010), though retrospective studies suggest that the magnitude of senile plaques is comparable between AD with and without diabetes (Kalaria, 2009). Several groups reported that a high-fat diet causes Aβ accumulation in the brains of wild type rabbits (Sparks et al., 1994) and APP Tg mice (Refolo et al., 2000; Ho et al., 2004). In a murine model of AD, inducing acute hyperglycemia increases Aβ production (Macauley et al., 2015). Moreover, altering insulin and insulin signaling may change Aβ levels in the brain through proteolysis by insulin-degrading enzymes (Vekrellis et al., 2000) and/or Aβ clearance from the brain (Shiiki et al., 2004). Alternative mechanisms might include the accumulation of autophagosomes to enhance amyloidogenic APP processing (Son et al., 2012) or up-regulation of BACE1 (Guglielmotto et al., 2012). APP^+^-*ob*/*ob* mice, generated by crossing diabetic *ob/ob* mice, display increased Aβ deposition in the cerebral vasculature (Takeda et al., 2010). Whether glycogen synthase kinase-3(GSK3) controls APP processing and Aβ levels in brain is intriguing (Phiel et al., 2003; Sereno et al., 2009; Sofola et al., 2010), but controversial (Jaworski et al., 2011). Anti-diabetic drug, pioglitazone, stimulated Aβ degradation by both microglia and astrocytes in ApoE-dependent manner (Mandrekar-Colucci et al., 2012). Thus, glucose metabolism is also involved in modulating Aβ levels.

## The role of glucose-energy metabolism in tau modulation

Several neuropathological studies suggest that the magnitude of NFTs in the brain at autopsy is not different between AD patients with and without diabetes (Kalaria, 2009). However, one report suggests that insulin resistance is associated with higher tau levels in the cerebrospinal fluid (Starks et al., 2015). Moreover, animal studies show that tau phosphorylation is increased in diabetes (Clodfelder-Miller et al., 2006; Jolivalt et al., 2008; Ke et al., 2009; Kim et al., 2009; Qu et al., 2011). For example, tau phosphorylation is increased in *db/db* mice (Kim et al., 2009), streptozotocin-treated wild type mice (Clodfelder-Miller et al., 2006; Jolivalt et al., 2008; Qu et al., 2011), and mutant human tau mice (Ke et al., 2009). Importantly, in humans, tau phosphorylation sites observed in AD are also increased in the diabetic brain (Liu et al., 2009). Conversely, CSF tau predicts changes in brain glucose metabolism, in turn causing longitudinal cognitive changes (Dowling et al., 2015). An energy-sensor, AMP-activated kinase (AMPK) activation is increased in the AD brain and AMPK phosphorylates Tau (Mairet-Coello et al., 2013). GSK3 also induces tau phosphorylation (Hanger et al., 1992; Yang et al., 1993). Anti-diabetic drug, metformin induces protein phosphatase 2A activity and reduces tau phosphorylation *in vitro* and in animal models (Kickstein et al., 2010). Therefore, glucose-energy metabolism is closely related to modulation of tau.

## The role of glucose-energy metabolism in neurodegeneration modulation

Diabetes causes structural deficits in the brain (Sato and Morishita, 2014) indicating that glucose-energy metabolism modulates neurodegeneration. Even in young adults, hyperglycemia is associated with subtle brain injury and impaired attention and memory (Weinstein et al., 2015). Indeed, diabetes reduces the volume of the hippocampus (Moran et al., 2013; Roberts et al., 2014), gray (García-Casares et al., 2014) and white matter (Moran et al., 2013). Gray matter loss occurs in the temporal, anterior cingulate, and frontal lobes (Moran et al., 2013; García-Casares et al., 2014; Roberts et al., 2014; Erus et al., 2015), while white matter loss appears in the frontal and temporal regions (Moran et al., 2013). In patients with AD, gray matter loss occurs in the temporal lobe, hippocampus, entorhinal and parietal lobes (Braak and Braak, 1991; Thompson et al., 2003; Andrade-Moraes et al., 2013), and white matter loss occurs in the temporal region (Mann, 1991). These studies indicate that diabetes causes neurodegeneration in the frontal and temporal lobes and other regions (Sato and Morishita, 2014). The molecular mechanism by which diabetes modulates neurodegeneration has not been fully elucidated, though several possible mechanisms have been proposed. Disturbance of glucose metabolism by GLUT1 deficiency causes neurodegeneration in APP Tg mice (Winkler et al., 2015). Another link between glucose hypometabolism and the progression of AD is the O-GlcNAcylation of proteins (Zhu et al., 2014). Decreased O-GlcNAcylation occurs in AD, which suggests that glucose hypometabolism may impair the protective roles of O-GlcNAc in neurons and lead to neurodegeneration (Zhu et al., 2014). AMPK is an energy-sensor, and AMPK over-activation is sufficient to cause dendritic spine loss (Mairet-Coello et al., 2013). Disturbed mTOR signaling affected by glucose-energy metabolism also causes neurodegeneration through mitochondrial dysfunction and autophagy (Perluigi et al., 2015). An observational study suggests that pioglitazone treatment is associated with a reduced dementia risk in diabetes patients (Heneka et al., 2015). Metformin is also reported to reduce the risk of cognitive decline in diabetes patients (Ng et al., 2014), though other group showed an opposite effect (Moore et al., 2013). These studies indicate that the molecular mechanism by which the dysregulation of glucose-energy metabolism causes neurodegeneration should be targeted to develop novel dementia therapies.

## Summary

Recent large, long-term, randomized controlled trials suggest that a multidisciplinary intervention, including exercise and diet, could improve or maintain cognitive function in at-risk elderly people (Ngandu et al., 2015). Exercise and diet alter glucose and lipid metabolism in subjects. As reviewed here, disruption of homeostasis of lipid and glucose metabolism affects production and clearance of β-amyloid and tau phosphorylation, and induces neurodegeneration. Therefore, a more integrated understanding of the interactions among lipid, glucose, and protein metabolism will be required to elucidate the pathogenesis of AD and to develop next-generation therapeutic options.

### Conflict of interest statement

The authors declare that the research was conducted in the absence of any commercial or financial relationships that could be construed as a potential conflict of interest.
